# Gamma-Ray Attenuation and Exposure Buildup Factor of Novel Polymers in Shielding Using Geant4 Simulation

**DOI:** 10.3390/ma14175051

**Published:** 2021-09-03

**Authors:** Mahmoud T. Alabsy, Jamila S. Alzahrani, M. I. Sayyed, Mahmoud I. Abbas, Daria I. Tishkevich, Ahmed M. El-Khatib, Mohamed Elsafi

**Affiliations:** 1 Physics Department, Faculty of Science, Alexandria University, Alexandria 21511, Egypt; mahmoud.alabsy@yahoo.com (M.T.A.); mabbas@physicist.net (M.I.A.); elkhatib60@yahoo.com (A.M.E.-K.); 2 Physics Department, College of Science, Princess Nourah Bint Abdulrahman University, Riyadh 11671, Saudi Arabia; jsalzahrani@pnu.edu.sa; 3 Department of Physics, Faculty of Science, Isra University, Amman 11622, Jordan; dr.mabualssayed@gmail.com; 4 Department of Nuclear Medicine Research, Institute for Research and Medical Consultations (IRMC), Imam Abdulrahman Bin Faisal University (IAU), P.O. Box 1982, Dammam 31441, Saudi Arabia; 5 Laboratory of Magnetic Films Physics, Scientific and Practical Materials Research, Centre of National Academy of Sciences of Belarus, 220072 Minsk, Belarus; dashachushkova@gmail.com; 6 Laboratory of Single Crystal Growth, South Ural State University, 454080 Chelyabinsk, Russia

**Keywords:** Geant4 simulation, radiation shielding, polymers, attenuation coefficients, XCOM software, exposure buildup factor and effective atomic number

## Abstract

Polymers are often used in medical applications, therefore, some novel polymers and their interactions with photons have been studied. The gamma-ray shielding parameters for Polymethylpentene (PMP), Polybutylene terephthalate (PBT), Polyoxymethylene (POM), Polyvinylidenefluoride (PVDF), and Polychlorotrifluoroethylene (PCTFE) polymers were determined using the Geant4 simulation and discussed in the current work. The mass attenuation coefficients (*μ*/*ρ*) were simulated at low and high energies between 0.059 and 1.408 MeV using different radionuclides. The accuracy of the Geant4 simulated results were checked with the XCOM software. The two different methods had good agreement with each other. Exposure buildup factor (EBF) was calculated and discussed in terms of polymers under study and photon energy. Effective atomic number (*Z_eff_*) and electron density (*N_eff_*) were calculated and analyzed at different energies. Additionally, the half-value layer (HVL) of the polymers was evaluated, and the results of this parameter showed that PCTFE had the highest probability of interaction with gamma photons compared to those of the other tested polymers.

## 1. Introduction

Nuclear technology is widely used in fields such as agriculture, medical applications, nuclear power plants, material identification, science, and space exploration. Researchers have developed and studied several new shielding materials [1,2,3,4] to safe humans from the hazards of ionizing radiation that originates from radioactive sources. Generally, the attenuation coefficients of gamma-rays characterize the interaction of radiation with the materials. It was observed that the absorption materials containing elements with high Z, or atomic numbers (such as Pb, Bi, and Ba) are utilized to attenuate the photons. However, medical applications often use materials containing low Z (such as H, C, N, and O).

Substances containing low Z (such as plastics and polymers) are commonly used as phantom materials and tissue equivalents in medical applications. In this case, linear attenuation coefficient, mass attenuation coefficient, effective atomic number, exposure buildup factor, and half- and tenth-value layers must be studied because they serve as indicators of the interactions of polymers with photons. The literature reports on the interaction between polymers with neutrons and/or gamma rays [5,6,7,8,9].

Polymehtylpentene (PMP) has useful properties, including excellent electrical insulator, extremely low water absorption, and good chemical resistance. In addition, it has high transparency, with a light transmittance of over 90%, which is equivalent to that of acrylic and glass. PMP also has a relatively high melting point of about 230 °C [10]. The second most important commercial polyester is Polybutylene terephthalate (PBT). PBT can easily be thermoformed and molded. Depending on the molding process and the cooling rate, it can be amorphous or semi-crystalline. PBT has some attractive properties, such as high durability and strength, heat resistance, good abrasion, excellent dimensional stability, and good chemical resistance, especially when reinforced with glass fiber [11].

Polyoxymethylene (POM), also called Acetal, is the most important Polyacetal. The crystalline thermoplastic is known for its high tensile and flexural strength, hardness, low creep under stress, and stiffness. It also has a low friction coefficient, excellent fatigue properties, and excellent chemical resistance, but has moderate thermal stability. Polyvinylidene fluoride (PVDF) is a highly inert specialty thermoplastic with a melting point of about 175 °C. It is produced by the radical polymerization of 1,1-difluoroethylene (CH2 = CF2) [10,11]. Polychlorotrifluoroethylene (PCTFE) is a transparent, non-flammable, semi-crystalline high-performance thermoplastic with excellent moisture resistance and high thermal resistance and chemical stability. It provides a balance of chemical, mechanical, and electrical properties, which is not available in any other engineered thermoplastics [10,11].

These characteristics of polymers make them important in the radiation industry. Understanding the interaction of these polymers with radiation will help applications that utilize radiation. The purpose of the current work is to evaluate the gamma-ray attenuation properties of the present polymers. Using a Geant4 simulation and the XCOM program [12,13], the shielding parameters of the polymers were determined to evaluate their attenuation characteristics against gamma-rays.

## 2. Materials and Methods

### 2.1. Shielding Parameters

The linear attenuation coefficient (*μ*) is a key factor for evaluating the effect of gamma radiation of appropriate energy with the studied material and can be deduced from Beer-Lambert Law [14] as follows in Equation (1):

(1) $$\mu =\frac{1}{x}\ln\left(\frac{I_{0}}{I}\right)=\frac{1}{x}\ln\left(\frac{Area_{without}}{Area_{with}}\right)$$

The initial and transmitted intensities are *I*_0_ and *I* respectively, across a target material of thickness *x*. *I*_0_ and *I* were determined by evaluating the area under the photopeak in without the polymer absorber *Area_without_* and with the polymer sample *Area_with_* respectively.

The ability of the considered polymers to be checked as radiation protecting materials by calculating the mass attenuation coefficient (*μ*/*ρ*) by dividing the calculated linear attenuation coefficient (*μ*) of a particular polymer by its density (*ρ*). Theoretically, (*μ*/*ρ*) can be evaluated using Equation (2) [15]:

(2) $$\frac{\mu }{\rho }=\underset{i}{\sum }w_{i}{\left(\frac{\mu }{\rho }\right)}_{i}$$

where *w_i_* and (*μ*/*ρ*)*_i_* was the weight fraction and the mass attenuation coefficient of the *i*th constituent element in the polymer material, respectively.

The half, tenth value layers or *HVL* and *TVL* are two important parameters in designing a suitable radiation shielding material. These parameters are defined as the attenuator thicknesses needed to decrease the γ-ray intensity to 50% and 10% of its initial value and estimated using Equations (3) and (4), respectively [16]:

(3) $$HVL=\frac{\ln2}{\mu }$$

(4) $$TVL=\frac{\ln10}{\mu }$$

Due to the interaction of gamma rays with the polymer sample, the mean-free path (MFP) is known as the medium distance traveled by a photon between two successive reactions is defined as and described in Equation (5) [17,18]:

(5) $$MFP=\frac{1}{\mu }$$

The *MFP* is also, practically, the attenuator distance which decreases the initial photon intensity of 36.8% when passing across the polymer absorber. The (*Z_eff_*) is another useful radiation interaction factor used to discuss the attenuating properties of the mixtures or compounds in terms of pure elements and depends on the incoming photon energy. *Z_eff_* values for the studied polymers can be obtained using Equation (6) [18]:

(6) $$Z_{eff}=\frac{\sum_{i}f_{i}A_{i}{\left(\frac{\mu }{\rho }\right)}_{i}}{\sum_{j}\frac{A_{j}}{Z_{j}}{\left(\frac{\mu }{\rho }\right)}_{j}}$$

where *f_i_*, *Z_i_* and *A_i_*, refer to the molar fraction, atomic number and atomic weight of the *i*th constituent element in the selected polymer, respectively.

The effective electron density (*N_eff_*), measured in electrons/g, defined as the number of electrons per unit mass of the polymer material and is derived using the calculated *Z_eff_* according to Equation (7) [19]:

(7) $$N_{eff}=\frac{N_{A}Z_{eff}}{\langle A\rangle }$$

where $\langle A\rangle =\underset{i}{\sum }f_{i}A_{i}$ represents the mean atomic mass of the polymer, and *N_A_* is Avogadro’s number.

When choosing a shielding material, the exposure buildup factor (EBF) must be considered to edit the absorption calculations resulting from buildup of secondary photons resulting from Compton scattering [20]. To determine the EBF for the selected polymers, the Geometric-Progression fitting method (GP) was employed, and the computations were determined according to the three following steps [7]:

The (*Z_eq_*), which is an energy-dependent parameter describing the properties of the investigated polymers in terms of their equivalent elements, was first calculated using the next formula [21,22]:

(8) $$Z_{eq}=\frac{Z_{1}\left(\log R_{2}-\log R\right)+Z_{2}\left(\log R-\log R_{1}\right)\;\;}{\log R_{2}-\log R_{1}}$$

where *R*_1_ and *R*_2_ are the (*μ_Comp_*/*μ_total_*) ratios corresponding to the elements with atomic numbers *Z*_1_ and *Z*_2_, respectively, and *R* is the (*μ_Comp_*/*μ_total_*) ratio for the selected polymer at a specific energy, which lies between the ratios *R*_1_ and *R*_2_.

The computed *Z_eq_* values of the investigated polymers were then used to interpolate the GP fitting EBFs (*b*, *c*, *a*, *X_K_*, *d*) in the range of energy 0.015–15 MeV using the interpolation formula [22] (9):

(9) $$C=\frac{C_{1}\left(\log Z_{2}-\log Z_{eq}\right)+C_{2}\left(\log Z_{eq}-\log Z_{1}\right)\;\;}{\log Z_{2}-\log Z_{1}}$$

where *C*_1_ and *C*_2_ are GP fitting parameters, taken from the ANSI/ANS-6.4.3 standard database [23], corresponding to *Z*_1_ and *Z*_2_ between which *Z_eq_* of the selected polymer lies. As an example, the GP fitting parameters and the *Z_eq_* for PMP (C_6_H_12_) in the energy range 0.015–15 MeV are listed in Table 1.

Finally, the EBF for the selected polymers were then estimated with the help of the obtained GP fitting parameters, using the following relations [24,25]:

(10) $$B\left(E,x\right)=1+\frac{b-1}{K-1}\;\left(K^{x}-1\right)\;,\;K\neq 1\;$$

and

(11) B(E,x)=1+(b−1)x , K=1

where

(12) $$K\left(E,x\right)=cx^{a}+d\frac{\tanh\left(x/X_{K}-2\right)-\tanh\left(-2\right)}{1-\tanh\left(-2\right)}\;\;\mathrm{for}\;x\;\leq \;40\;\mathrm{mfp}$$

where *E* is incident γ-ray energy and *x* is the penetration depth in terms of mfp.

### 2.2. Geant4 Simulation

Geant4 toolkit is a universal Monte Carlo code that can be used to study the history of the electron, neutron and photon or their coupling within a medium. With its huge cross-section database, Geant4 can simulate the interaction between these particles and energies from 1 keV to 100 MeV [26]. The input of the detector form will be explained as a Monte Carlo symbol and the calculated physical quantity. Geant4 was divided into different sizes, and the largest was called the "universal size". The simulation is set up inside this folder. 

**Figure 1:**
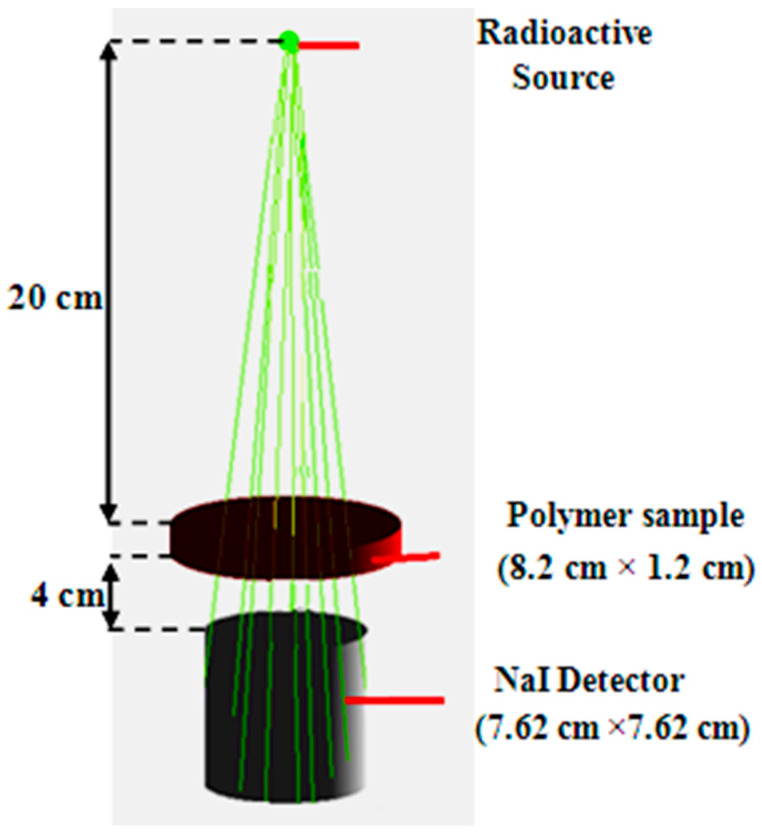
Sketch of the used Geant4 simulation geometry set-up.

Within the large volume “world”, the design and construction of the detector, attenuating material, and radioactive source are shown in Figure 1. The detector was modeled with a NaI crystal, which was cylindrical and surrounded by aluminum housing (0.5 mm), and the reflective material was modeled with magnesium oxide. For the attenuation material, it was modeled by using different attenuation materials in the simulation process, and different polymer materials with a constant thickness were placed between the source and the detector. There are two methods for modeling radioactive sources: the first method is to use radionuclides, which is necessary to understanding the decay scheme of each radioactive source, and the second method is the method used in a recent article, [27], using monoenergetic gamma-ray photons in each simulation.

**Figure 2:**
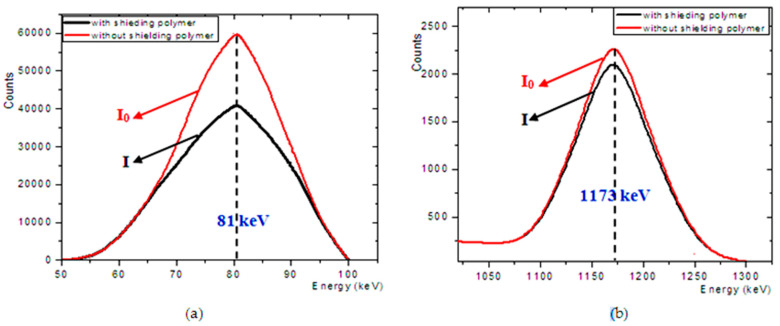
The energy spectra from shielded and unshielded simulations at different two energies (**a**) at 81 and (**b**) 1173 keV.

For this purpose, the Gaussian distribution of the parameters (standard deviation σ and mean x) obtained from the analytical data was utilized to the deposition energy E_0_ [27,28,29]. First, the full width at half maximum (FWHM) on the energy deposited (E_0_) was evaluated. The second step was to create random energy or (E_r_) by using a Gaussian distribution with mean <x> = E_0_ and σ = 2.356 * FWHM (E_0_)). If it was a random in the interval (E_0_ ± 2.96 σ), then the peak value was energy E_0_. In the ROOT analysis framework, an algorithm written in C++ was used to process the Gaussian distribution spectrum [30]. The algorithm performed a peak search process and calculated the area under the peak using the same mathematical algorithm as in Genie2000 [31] (see Figure 2). The weighted peak area with and without attenuating material at different energies was obtained (see Table 2) and used in Equation (1) to calculate the attenuation coefficient of present polymer materials. To reduce the error within 1%, no less than 2 × 10^7^ events were used in each simulation, otherwise, the error would increase as the number decreased.

## 3. Results and Discussion

The Geant4 MC code was utilized to estimate the mass attenuation coefficients (*μ*/*ρ*) of the five polymer samples at energies between 0.05 and 1.4 MeV. Table 2 lists the results from Geant4 and the XCOM program and the corresponding relative differences. It is evident from Table 2 that under the selected γ-ray energy, the simulated *μ*/*ρ* results of the polymer samples were quite close to the theoretical results of the XCOM code at all energy regions. Therefore, the estimated *μ*/*ρ* value of the polymer showed the same dependence on γ-ray energy. The relative deviation RD (%) between XCOM and Geant4 values was computed using Equation (13):

(13) $$RD\left(\%\right)=\frac{{\left(\mu /\rho \right)}_{XCOM}-{\left(\mu /\rho \right)}_{Geant4}}{{\left(\mu /\rho \right)}_{XCOM}}\times 100$$

**Figure 3:**
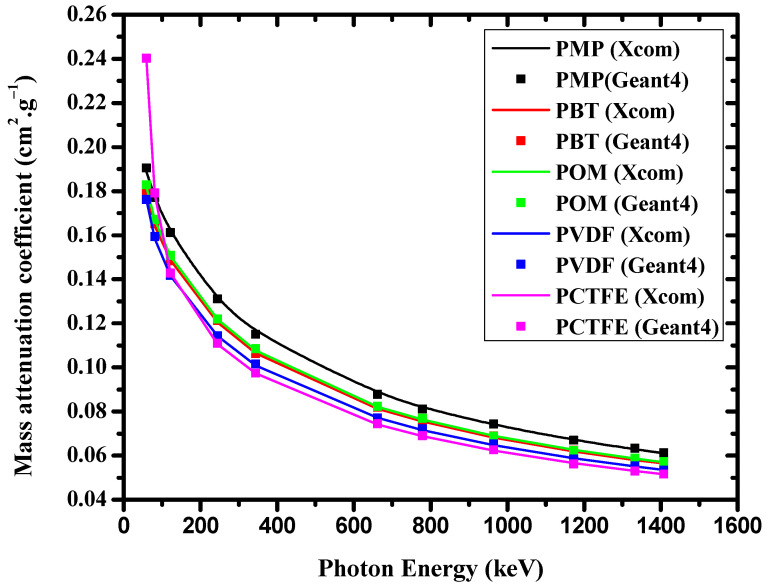
Mass attenuation coefficients of PMP, PBT, POM, PVDF, and PCTFE polymers as a function of photon energy from XCOM and Geant4.

The *μ*/*ρ* values obtained using the XCOM online database of photon interaction cross-sections are plotted in Figure 3 relative to the Geant4 data with γ-ray energy in the range of 0.059–1.408 MeV. This figure clarifies that PCTFE’s value of *μ*/*ρ* was the highest at low energies and the lowest at high energies. In addition, it can be seen that among all the polymer types, the maximum *μ*/*ρ* value occurred at low energies, while the lowest *μ*/*ρ* occurred at high energies.

**Figure 4:**
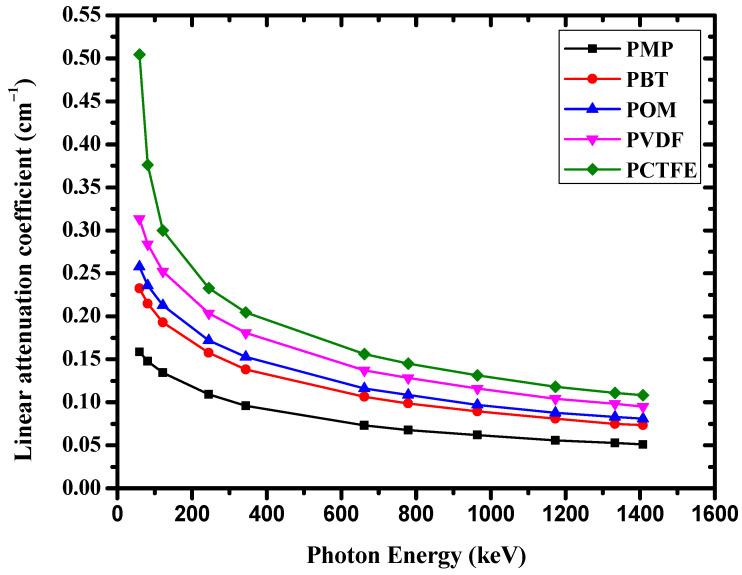
Geant4 linear attenuation coefficients of the present polymers as a function of photon energy.

It is obvious from Figure 4 that as the photon energy increased, the μ value dropped sharply. This behavior can be attributed to the probability of interaction at low energy, where the predominant interaction is the photoelectric effect and its cross-section approximately increasing with E^−3.5^. The dominant phenomenon at the medium γ-ray energy region is known Compton scattering and its cross-section changes with E^−1^ and Z. While, in the high-energy, Compton scattering and the pair production process have a mixed effect.

**Figure 5:**
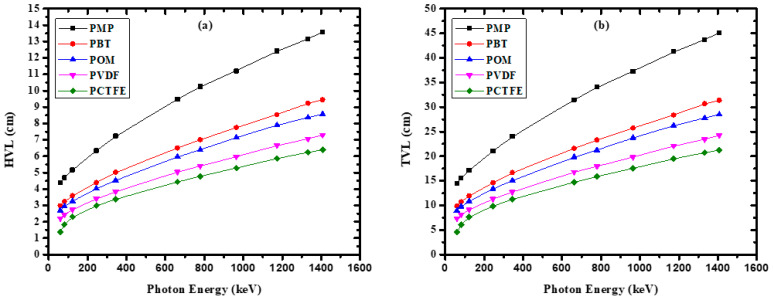
The variation of (**a**) half-value layer and (**b**) tenth-value layer with photon energy for the investigated polymers.

The variation of HVL and TVL values of the present polymers as a function of photon energies is depicted in Figure 5. According to Figure 5, PCTFE had the lowest HVL value, ranging from 2 to 6 cm, while PMP had the highest HVL value, ranging from 4.5 to 13.55 cm. These results imply that the higher the polymer density, the lower the HVL value, which enhances the shielding ability of the chosen polymer. Figure 5 also demonstrates that all the polymer samples had the lowest HVL results at low γ-ray energies. This trend may be due to the advantage of the photoelectric effect in this energy region. As the photon energy increased to the medium-energy region, the HVL value could be observed to gradually increase. This increase occurred because of the dominance of Compton scattering in this region. The little increase in HVL results at higher energy could be attributed to the dominance of the pair production process at these energies.

**Figure 6:**
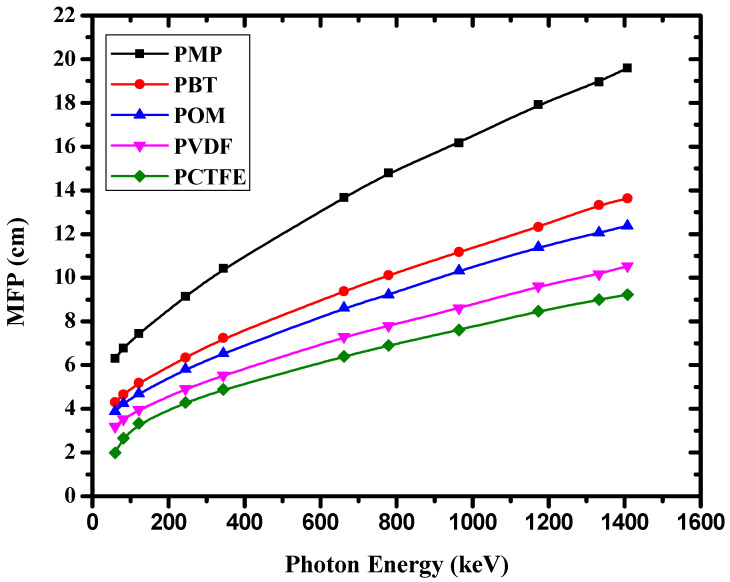
The variation of mean-free path with increasing photon energy for the investigated polymers.

Figure 6 shows that the MFP values of the investigated polymer varied with γ-ray energy in the range of 0.059–1.408 MeV. From Figure 6, the γ-ray energy dependence of the MFP value was similar to the HVL trend, so the same discussion about photon interactions applies. The MFP values decreased from POM to PCTFE, which indicates that compared to other polymers, photons in this energy range could penetrate POM to the greatest extent.

**Figure 7:**
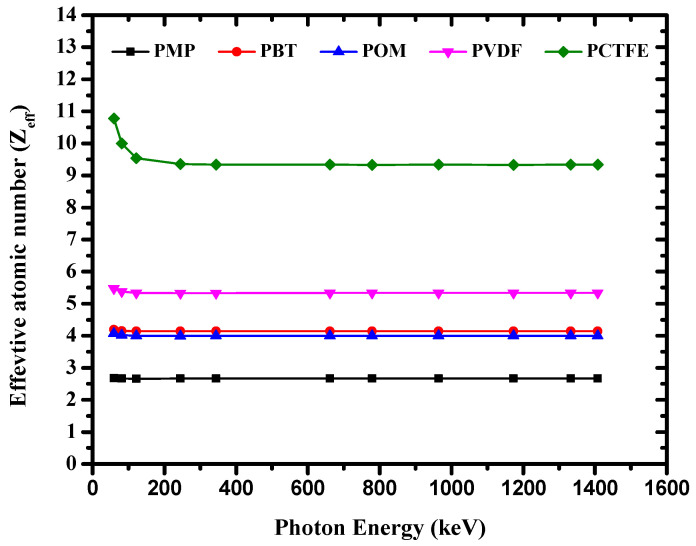
The variation of *Z_eff_* with photon energy for the investigated polymers.

**Figure 8:**
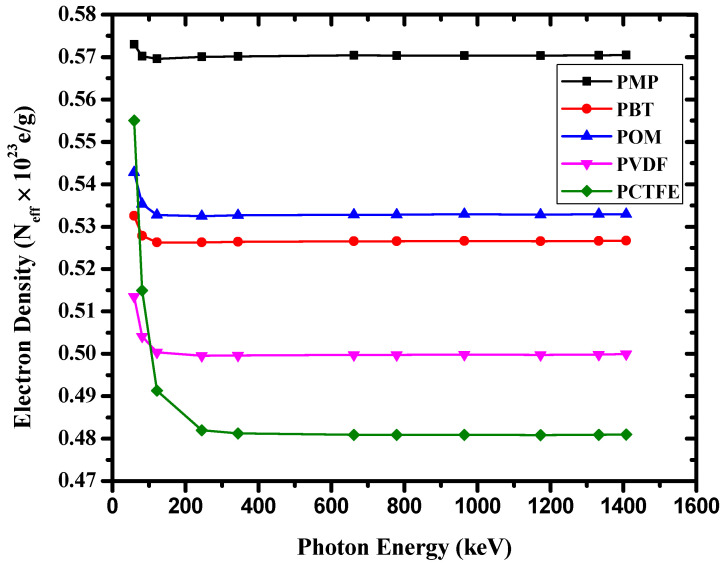
The variation of *N_eff_* with γ-ray energy for the investigated polymers.

The *Z_eff_* of the studied materials was evaluated from the calculated (*μ*/*ρ*). The results were obtained and are shown in Figure 7. Figure 7 shows that the *Z_eff_* of PCTFE was greater than that of other discussed polymers. This high value was mainly due to the contents of fluorine (F) and chlorine (Cl) in PCTFE, besides the hydrogen content, which was negligible. Similarly, Figure 7 shows that *Z_eff_* of PBT and POM were nearly the same, but relatively lower than that of PVDF. Among the studied polymers, POM had the lowest *Z_eff_* value because of the high hydrogen content in this polymer. It is obvious from Figure 7 that *Z_eff_* values for all polymers remained almost constant. The *Z_eff_* trend of these five polymers was occurring because the present polymers were composed of elements very close in atomic numbers (such as H, C, O, and N). The changes of *Z_eff_* and *N_eff_* with photon energy are shown in Figure 7 and Figure 8, respectively.

**Figure 9:**
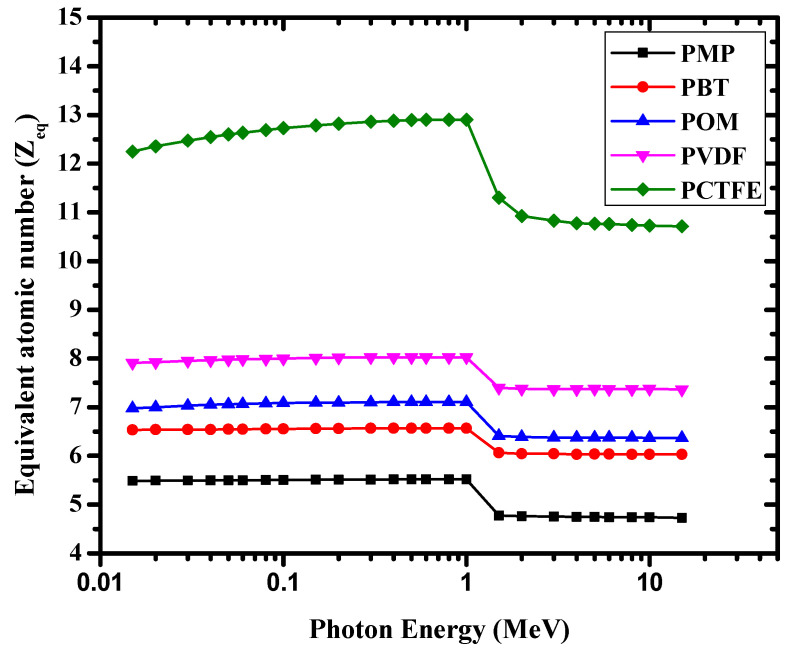
Z_eq_ variation with different energy of photons for the investigated polymers.

**Figure 10:**
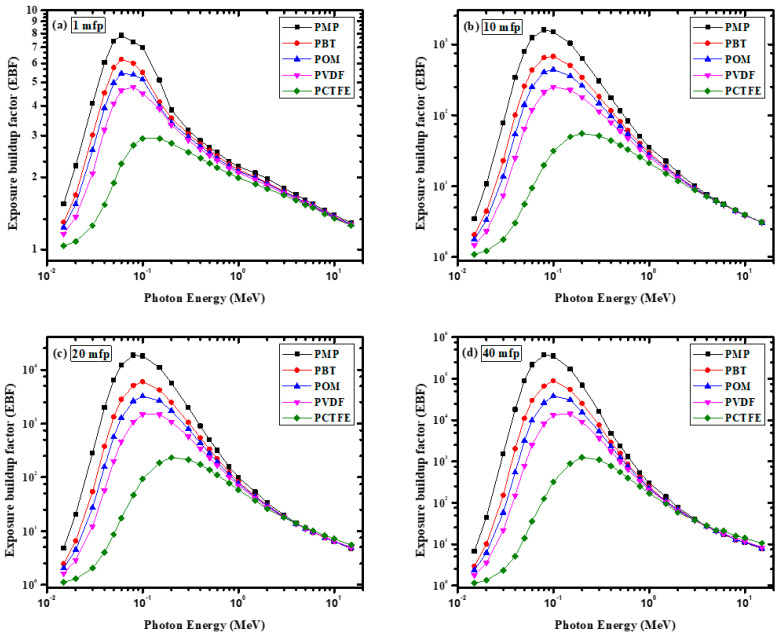
The variation of EBF with γ-ray energy for the investigated polymers at (**a**) 1 mfp, (**b**) 10 mfp, (**c**) 20 mfp, and (**d**) 40 mfp.

Figure 9 shows the calculated Zeq values for these polymers. The GP fitting parameters of PCTFE are tabulated in Table 1. The EBF of a given polymer varied between 0.015 and 15 MeV with photon energy. Additionally, Figure 10 illustrates the EBF of the samples at a fixed penetration depth. A similar trend has been studied for different samples reported in the reference [33]. The EBF values in the form demonstrate that as the energy increased, the values increased up to the medium-energy area. In addition, in the medium-energy region, at 10 and 40 mfp, the EBF value appeared to be very large. This result was due to the Compton scattering process at this energy. In this region, the γ-ray photons are not completely extracted, but their energies are reduced. These photons existed in the sample for a long time, causing multiple Compton scattering interactions, which raised the EBF to a higher value between 0.08 and 0.2 MeV.

**Figure 11:**
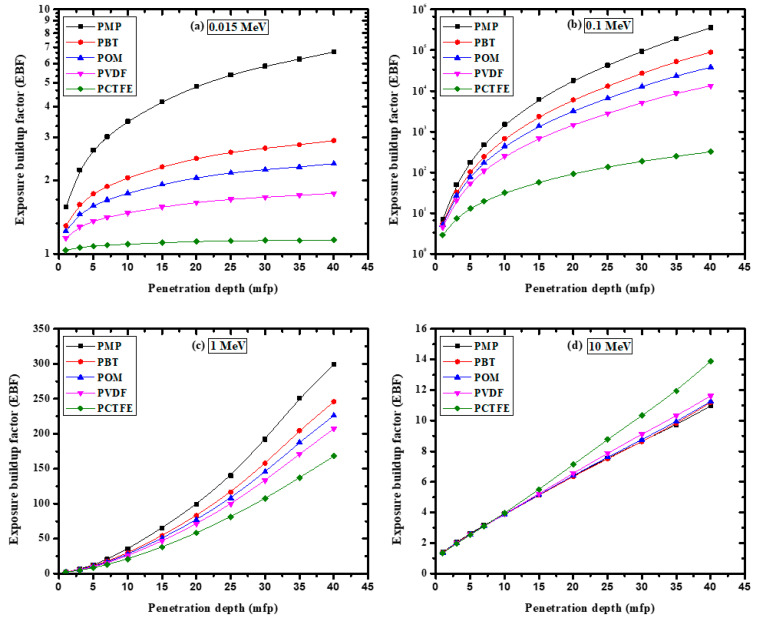
The variation of the exposure buildup factor with penetration depth for the tested polymers at (**a**) 0.015 MeV, (**b**) 0.1 MeV, (**c**) 1 MeV, and (**d**) 10 MeV.

**Figure 12:**
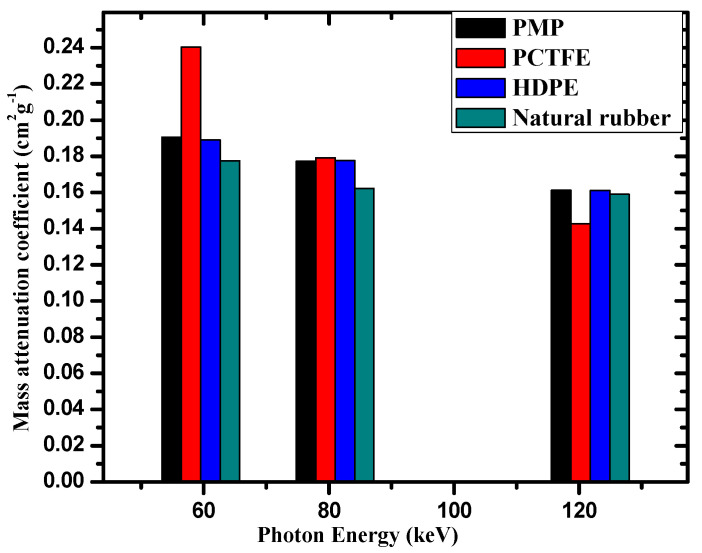
Mass attenuation coefficients of PMP and PCTFE polymers compared to other discussed experimental values of HDPE and natural rubbers.

The EBF results for all the studied polymer samples had approximately the same value between 4 and 15 MeV, indicating that EBF had no relationship with the composition of the discussed samples in this region. The EBF trend between 4 and 15 MeV was due to the pair production process, a very important photon interaction phenomenon at these high energies. Additionally, among the selected polymers, POM had the highest EBF value. Likewise, PCTFE had the lowest EBF value. The lower EBF values for PCTFE could be attributed to the higher Zeq of this polymer. Moreover, it was found from Figure 10 that the EBF value of the polymer increased with increasing penetration depth. The lowest EBF value for all the samples was found at 1 mfp (Figure 10a), while the highest EBF value at 40 mfp (Figure 10d). This result can be attributed to multiple dispersions at a large penetration depth. In other words, an increase in the depth of penetration led to an increase in the photon interaction with the sample (see Figure 11), which led to the generation of low-energy photons. Finally, the mass attenuation coefficients of the present polymers were compared to those from some recent reports on HDPE [3] and natural rubber [34]. The comparison is shown in Figure 12 and shows a difference between them in the low energies, while the results are similar in the high energies.

## 4. Conclusions

The photon attenuation characteristics of various studied polymers (such as PMP, PBT, POM, PVDF, and PCTFE) were studied using factors such as HVL, MFP, *Zeff*, and EBF. A Geant4 simulation was used to determine these parameters at energies between 59 and 1408 keV. The maximum linear attenuation coefficient was determined for the PCTFE polymer. PCTFE’s *Z_eff_* was found to be relatively higher than that of other polymers. The HVL and MFP value of PCTFE were also lower than the value for other polymers, which indicates that this polymer attenuated gamma-rays more effectively. The results demonstrated that out of the present polymers, PMP’s EBF values tended to be high while PCTFE’s EBF tended to be low. The EBF values of the present polymers increased with penetration depth, being highest for 40 mfp and lowest for 1 mfp. This paper concludes that the current polymers can be used alone in low-energy or in combination with other high-Z materials for high-energy gamma-ray shielding.

## Figures and Tables

**Table 1:** The (*Z_eq_*) and GP fitting parameters for PMP (C_6_H_12_) in the range 0.015–15 MeV.

Energy (MeV)	*Z_eq_*	b	c	a	X_K_	d
0.015	5.488	1.553	0.610	0.121	14.66	−0.059
0.02	5.494	2.240	0.842	0.051	15.20	−0.029
0.03	5.494	4.085	1.400	−0.070	13.62	0.029
0.04	5.495	6.053	1.985	−0.156	14.25	0.068
0.05	5.499	7.443	2.371	−0.195	14.40	0.085
0.06	5.501	7.862	2.652	−0.221	14.45	0.097
0.08	5.505	7.391	2.917	−0.244	14.54	0.106
0.1	5.506	7.007	2.884	−0.239	15.18	0.100
0.15	5.510	5.111	2.966	−0.251	14.72	0.106
0.2	5.512	3.832	2.942	−0.254	14.64	0.114
0.3	5.514	3.151	2.652	−0.236	14.21	0.110
0.4	5.516	2.852	2.397	−0.216	13.06	0.095
0.5	5.516	2.667	2.179	−0.195	13.49	0.091
0.6	5.516	2.545	1.994	−0.173	13.64	0.080
0.8	5.517	2.334	1.784	−0.149	13.61	0.075
1	5.517	2.222	1.604	−0.122	13.66	0.062
1.5	4.779	2.090	1.408	−0.091	13.62	0.053
2	4.762	1.978	1.229	−0.054	14.18	0.031
3	4.754	1.803	1.073	−0.019	12.81	0.012
4	4.749	1.697	0.980	0.005	15.34	−0.003
5	4.747	1.609	0.933	0.018	15.48	−0.010
6	4.745	1.551	0.900	0.027	14.82	−0.015
8	4.744	1.455	0.868	0.036	14.27	−0.020
10	4.742	1.396	0.834	0.048	13.62	−0.025
15	4.726	1.291	0.823	0.050	13.58	−0.024

**Table 2:** Comparison between mass attenuation coefficients for the polymer materials using XCOM software online [32] and Geant4 code at various photon energies.

Sample	Energy (keV)	Area without	Uncert.	Area with	Uncert.	Linear Attenuation Coefficient Geant4 (cm^−1^)	Mass Attenuation Coefficient (cm^2^ g^−1^)		
							Geant4	XCOM	Δ%
**PMP**	59.53	1.50 × 10^5^	6.71 × 10^2^	1.09 × 10^5^	5.72 × 10^2^	0.15866	0.19047	0.18880	0.88%
	80.99	1.67 × 10^5^	7.07 × 10^2^	1.24 × 10^5^	6.10 × 10^2^	0.14755	0.17713	0.17690	0.13%
	121.78	1.71 × 10^5^	7.17 × 10^2^	1.31 × 10^5^	6.26 × 10^2^	0.13428	0.16120	0.16070	0.31%
	244.69	1.52 × 10^5^	6.76 × 10^2^	1.22 × 10^5^	6.05 × 10^2^	0.10924	0.13114	0.13040	0.56%
	344.30	1.19 × 10^5^	5.98 × 10^2^	9.80 × 10^4^	5.46 × 10^2^	0.09583	0.11504	0.11520	−0.14%
	661.66	5.98 × 10^4^	4.24 × 10^2^	5.17 × 10^4^	4.20 × 10^2^	0.07317	0.08784	0.08802	−0.21%
	778.90	5.08 × 10^4^	3.91 × 10^2^	4.44 × 10^4^	3.65 × 10^2^	0.06759	0.08114	0.08174	−0.74%
	964.13	4.09 × 10^4^	3.51 × 10^2^	3.62 × 10^4^	3.30 × 10^2^	0.06185	0.07425	0.07387	0.51%
	1173.23	3.33 × 10^4^	3.17 × 10^2^	2.98 × 10^4^	3.23 × 10^2^	0.05577	0.06695	0.06708	−0.19%
	1332.50	3.02 × 10^4^	3.01 × 10^2^	2.72 × 10^4^	2.86 × 10^2^	0.05273	0.06330	0.06283	0.75%
	1408.01	2.78 × 10^4^	2.89 × 10^2^	2.51 × 10^4^	2.75 × 10^2^	0.05101	0.06123	0.06107	0.27%
**PBT**	59.53	1.50 × 10^5^	6.71 × 10^2^	9.42 × 10^4^	8.20 × 10^2^	0.23234	0.17873	0.17750	0.69%
	80.99	1.67 × 10^5^	7.07 × 10^2^	1.08 × 10^5^	5.77 × 10^2^	0.21465	0.16512	0.16420	0.56%
	121.78	1.71 × 10^5^	7.17 × 10^2^	1.16 × 10^5^	5.91 × 10^2^	0.19281	0.14831	0.14860	−0.19%
	244.69	1.52 × 10^5^	6.76 × 10^2^	1.11 × 10^5^	6.19 × 10^2^	0.15771	0.12132	0.12040	0.75%
	344.30	1.19 × 10^5^	5.98 × 10^2^	9.01 × 10^4^	5.67 × 10^2^	0.13814	0.10626	0.10640	−0.13%
	661.66	5.98 × 10^4^	4.24 × 10^2^	4.83 × 10^4^	4.31 × 10^2^	0.10656	0.08197	0.08128	0.84%
	778.90	5.08 × 10^4^	3.91 × 10^2^	4.17 × 10^4^	3.54 × 10^2^	0.09886	0.07604	0.07548	0.74%
	964.13	4.09 × 10^4^	3.51 × 10^2^	3.42 × 10^4^	3.21 × 10^2^	0.08948	0.06883	0.06823	0.88%
	1173.23	3.33 × 10^4^	3.17 × 10^2^	2.83 × 10^4^	3.38 × 10^2^	0.08114	0.06242	0.06195	0.75%
	1332.50	3.02 × 10^4^	3.01 × 10^2^	2.60 × 10^4^	3.29 × 10^2^	0.07504	0.05772	0.05803	−0.54%
	1408.01	2.78 × 10^4^	2.89 × 10^2^	2.40 × 10^4^	2.86 × 10^2^	0.07338	0.05644	0.05641	0.06%
**POM**	59.53	1.50 × 10^5^	6.71 × 10^2^	8.95 × 10^4^	5.42 × 10^2^	0.25771	0.18277	0.18180	0.53%
	80.99	1.67 × 10^5^	7.07 × 10^2^	1.04 × 10^5^	6.54 × 10^2^	0.23569	0.16716	0.16690	0.15%
	121.78	1.71 × 10^5^	7.17 × 10^2^	1.12 × 10^5^	5.79 × 10^2^	0.21264	0.15081	0.15040	0.27%
	244.69	1.52 × 10^5^	6.76 × 10^2^	1.07 × 10^5^	5.68 × 10^2^	0.17199	0.12198	0.12180	0.14%
	344.30	1.19 × 10^5^	5.98 × 10^2^	8.75 × 10^4^	5.70 × 10^2^	0.15292	0.10845	0.10760	0.78%
	661.66	5.98 × 10^4^	4.24 × 10^2^	4.74 × 10^4^	4.12 × 10^2^	0.11603	0.08229	0.08222	0.08%
	778.90	5.08 × 10^4^	3.91 × 10^2^	4.09 × 10^4^	3.67 × 10^2^	0.10849	0.07694	0.07636	0.76%
	964.13	4.09 × 10^4^	3.51 × 10^2^	3.37 × 10^4^	3.18 × 10^2^	0.09693	0.06875	0.06903	−0.41%
	1173.23	3.33 × 10^4^	3.17 × 10^2^	2.80 × 10^4^	2.90 × 10^2^	0.08779	0.06226	0.06266	−0.64%
	1332.50	3.02 × 10^4^	3.01 × 10^2^	2.56 × 10^4^	2.97 × 10^2^	0.08290	0.05879	0.05871	0.14%
	1408.01	2.78 × 10^4^	2.89 × 10^2^	2.37 × 10^4^	2.67 × 10^2^	0.08084	0.05733	0.05706	0.47%
**PVDF**	59.53	1.50 × 10^5^	6.71 × 10^2^	8.01 × 10^4^	5.55 × 10^2^	0.31363	0.17620	0.17600	0.11%
	80.99	1.67 × 10^5^	7.07 × 10^2^	9.44 × 10^4^	5.33 × 10^2^	0.28375	0.15941	0.15830	0.70%
	121.78	1.71 × 10^5^	7.17 × 10^2^	1.03 × 10^5^	5.57 × 10^2^	0.25233	0.14176	0.14150	0.18%
	244.69	1.52 × 10^5^	6.76 × 10^2^	1.01 × 10^5^	5.81 × 10^2^	0.20345	0.11430	0.11420	0.09%
	344.30	1.19 × 10^5^	5.98 × 10^2^	8.27 × 10^4^	5.80 × 10^2^	0.18075	0.10155	0.10090	0.64%
	661.66	5.98 × 10^4^	4.24 × 10^2^	4.55 × 10^4^	4.21 × 10^2^	0.13717	0.07706	0.07710	−0.05%
	778.90	5.08 × 10^4^	3.91 × 10^2^	3.93 × 10^4^	3.43 × 10^2^	0.12829	0.07207	0.07161	0.64%
	964.13	4.09 × 10^4^	3.51 × 10^2^	3.24 × 10^4^	3.36 × 10^2^	0.11622	0.06529	0.06473	0.86%
	1173.23	3.33 × 10^4^	3.17 × 10^2^	2.71 × 10^4^	2.85 × 10^2^	0.10403	0.05844	0.05877	−0.56%
	1332.50	3.02 × 10^4^	3.01 × 10^2^	2.48 × 10^4^	2.73 × 10^2^	0.09828	0.05522	0.05507	0.26%
	1408.01	2.78 × 10^4^	2.89 × 10^2^	2.30 × 10^4^	2.96 × 10^2^	0.09495	0.05334	0.05353	−0.35%
**PCTFE**	59.53	1.50 × 10^5^	6.71 × 10^2^	5.46 × 10^4^	4.44 × 10^2^	0.50455	0.24026	0.23990	0.15%
	80.99	1.67 × 10^5^	7.07 × 10^2^	7.85 × 10^4^	5.47 × 10^2^	0.37610	0.17910	0.17830	0.44%
	121.78	1.71 × 10^5^	7.17 × 10^2^	9.40 × 10^4^	5.31 × 10^2^	0.29980	0.14276	0.14280	−0.03%
	244.69	1.52 × 10^5^	6.76 × 10^2^	9.51 × 10^4^	5.34 × 10^2^	0.23285	0.11088	0.11050	0.34%
	344.30	1.19 × 10^5^	5.98 × 10^2^	7.89 × 10^4^	5.16 × 10^2^	0.20456	0.09741	0.09729	0.12%
	661.66	5.98 × 10^4^	4.24 × 10^2^	4.38 × 10^4^	3.88 × 10^2^	0.15624	0.07440	0.07420	0.27%
	778.90	5.08 × 10^4^	3.91 × 10^2^	3.80 × 10^4^	3.38 × 10^2^	0.14501	0.06905	0.06891	0.21%
	964.13	4.09 × 10^4^	3.51 × 10^2^	3.15 × 10^4^	3.23 × 10^2^	0.13135	0.06255	0.06229	0.41%
	1173.23	3.33 × 10^4^	3.17 × 10^2^	2.63 × 10^4^	3.04 × 10^2^	0.11807	0.05622	0.05655	−0.58%
	1332.50	3.02 × 10^4^	3.01 × 10^2^	2.42 × 10^4^	2.90 × 10^2^	0.11104	0.05288	0.05301	−0.25%
	1408.01	2.78 × 10^4^	2.89 × 10^2^	2.24 × 10^4^	2.99 × 10^2^	0.10836	0.05160	0.05154	0.12%

## Data Availability

All data are available in the manuscript.
